# Quartz Crystal Microbalance Frequency Response to Discrete Adsorbates in Liquids

**DOI:** 10.1021/acs.analchem.4c00968

**Published:** 2024-06-21

**Authors:** Alexander M. Leshansky, Boris Y. Rubinstein, Itzhak Fouxon, Diethelm Johannsmann, Marta Sadowska, Zbigniew Adamczyk

**Affiliations:** † Department of Chemical Engineering, Technion, Haifa 32000, Israel; ‡ Stowers Institute for Medical Research, 1000 East 50th Street, Kansas City, Missouri 64110, United States; § Institute of Physical Chemistry, Clausthal University of Technology, Arnold-Sommerfeld-Straße 4, 38678 Clausthal-Zellerfeld, Germany; ∥ Jerzy Haber Institute of Catalysis and Surface Chemistry, Polish Academy of Sciences, 30-239 Krakow, Poland

## Abstract

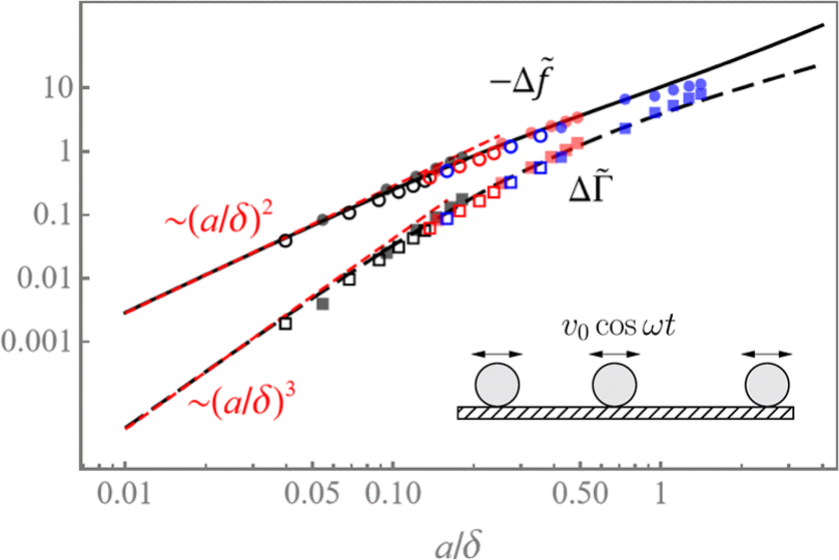

Quartz crystal microbalance with dissipation monitoring (QCM-D) has become a major tool enabling accurate investigation of the adsorption kinetics of nanometric objects such as DNA fragments, polypeptides, proteins, viruses, liposomes, polymer, and metal nanoparticles. However, in liquids, a quantitative analysis of the experimental results is often intricate because of the complex interplay of hydrodynamic and adhesion forces varying with the physicochemical properties of adsorbates and functionalized QCM-D sensors. In the present paper, we dissect the role of hydrodynamics for the analytically tractable case of stiff contact, whereas the adsorbed rigid particles oscillate with the resonator without rotation. Under the assumption of the low surface coverage, we theoretically study the excess shear force exerted on the resonator, which has two contributions: (i) the fluid-mediated force due to flow disturbance created by the particle and (ii) the force exerted on the particle by the fluid and transmitted to the sensor via contact. The theoretical analysis enables an accurate interpretation of the QCM-D impedance measurements. It is demonstrated inter alia that for particles of the size comparable with protein molecules, the hydrodynamic force dominates over the inertial force and that the apparent mass derived from QCM independently of the overtone is about 10 times the Sauerbrey (inertial) mass. The theoretical results show excellent agreement with the results of experiments and advanced numerical simulations for a wide range of particle sizes and oscillation frequencies.

## Introduction

Quartz crystal microbalance (QCM) technique^1,2^ relies on the fact that matter adsorbed on the surface of the fast oscillating crystal changes the frequency of the oscillations. In vacuum, the shift in the resonant frequency of the crystal is linearly proportional to the mass of the adsorbed film via the seminal Sauerbrey equation,^3^ allowing extremely accurate measurements down to nanograms.^2^ The quantitative interpretation of the QCM-D measurement in liquids^4,5^ (where “D” stands for dissipation monitoring via probing the decay rate of the oscillations) is also well-established for planar adsorbed (including viscoelastic) films,^6−9^ although the challenges with regards to application of the standard model for the planar layered systems still remain.

Interpreting the QCM-D measurements due to discrete adsorbates (such as, e.g., nanoparticles, liposomes, viruses, proteins, and so forth) in liquids remains a challenge mainly due to the interplay of complex hydrodynamics, which has not yet been yet fully resolved and a priori unknown viscoelastic contact dynamics, which depends on physicochemical properties of the surfaces (i.e., the adsorbate and the resonator).^2^ The experimental observations showing the considerable deviation from the Sauerbry equation due to discrete adsorbates are known since the early days of liquid-phase QCM and the “trapped liquid/solvent” hypothesis and the corresponding ad hoc phenomenological models were put forward to explain the apparent discrepancy in the mass of the adsorbates probed by the QCM.^10−12^

The impedance  probed by the QCM-D is the ratio , where  is the area-averaged tangential stress (i.e., the net shear force  exerted on the surface of the oscillating quartz resonator divided by its surface area) and *v*_c_ is the velocity of the crystal oscillations. Here,  and *v*_c_ and, therefore,  are all complex quantities characterized by the amplitude and phase. In the framework of the small load approximation, the shift in oscillation frequency, Δ*f*, and in half-bandwidth, ΔΓ (related to a dissipation factor ), are linearly proportional to the impedance, , where *f*_0_ stands for the fundamental oscillation frequency (typically 5 MHz) and the resonator’s shear-wave impedance  is a known quantity.^1,2^ In liquids, the small load approximation holds given that  In liquids, in contrast to vacuum where the rigidly attached particles only alter the mass (i.e., solid inertia) of the resonator contributing to the frequency shift, Δ*f*, according to the Sauerbrey equation, the discrete adsorbates modify the viscous shear force exerted onto the sensor, contributing to the shifts in the resonant frequency, Δ*f*, and the bandwidth, ΔΓ (absent in vacuum).

**Figure 1:**
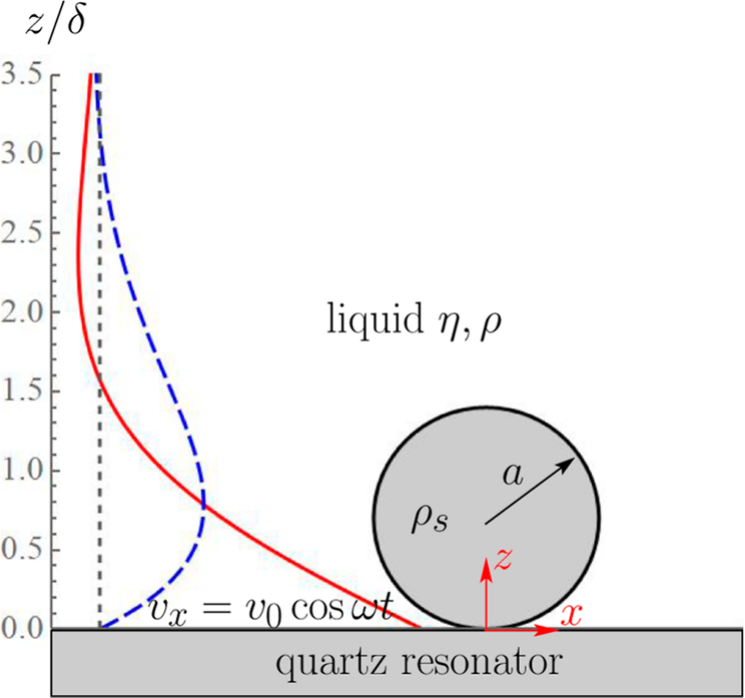
Schematic illustration of the problem. A spherical particle of radius *a* and density ρ_s_, immersed in an incompressible viscous liquid of density ρ and viscosity η, is rigidly attached to an infinite horizontal plane at *z* = 0 oscillating at MHz frequency with velocity ***v*** = *v*_0_***x̂*** cos ω*t*. The undisturbed (i.e., in the absence of the particle) velocity profiles, ***u***_0_ = *v*_0_***x̂*** Re[e^–*z*/δ^e^–i(ω*t*–*z*/δ)^], are shown at two time instants ω*t* = 0 (solid, red) and ω*t* = π/2 (dashed, blue) vs the scaled vertical distance *z*/δ. The short-dashed vertical line stands for the zero value of the velocity.

In the absence of particles, the horizontal small-amplitude time-periodic oscillations of the resonator at *z* = 0 with velocity  create unidirectional oscillatory flow of the viscous liquid of viscosity η and density ρ occupying the upper half-space *z* > 0 with velocity given by the real part of .^13^ The flow disturbance propagates upward as the transverse wave is attenuated by the exponential factor with δ = (2ν/ω)^1/2^ known as viscous penetration depth, where ν = η/ρ stands for the kinematic viscosity of the fluid (see Figure 1). Computing the shear stress at the resonator, , and dividing by the resonator velocity readily yields the impedance ,^5^ corresponding to a negative frequency shift and positive dissipation factor (as compared to the unloaded resonator oscillating in vacuum). Obviously, the particles located above the resonator would perturb this flow and modify the shear stress exerted on the resonator. The contribution to impedance due to the flow disturbance is entirely fluid-mediated, i.e., it takes place for both adsorbed and freely suspended particles as it does not require a physical contact between the particle and the resonator. For the adsorbed particle, however, there is another contribution to impedance due to the force exerted on it by the (perturbed) flow and transmitted to the resonator via contact.

The prior works applied a variety of numerical methods to account for the hydrodynamics and compute the perturbed viscous stress exerted on the resonator due to an adsorbed particle. Various factors, such as particle size, surface coverage, particle mobility (e.g., rocking vs sliding motion), deviation from sphericity, and other factors, were considered using finite element method (FEM) in the early works^14,15^ and later with lattice Boltzmann method^16−19^ and the immersed boundary method (IBM).^20,21^ Although the numerical methods are very powerful, the complex interplay of various factors and uncertainty of physicochemical properties and/or parameters governing the contact dynamics call for a more analytical approach able to dissect the role of the hydrodynamic forces in QCM-D analysis of discrete adsorbates. In ref (22) the hydrodynamic contribution to the impedance due to an adsorbed particle was approximated by the analytical result for the force exerted on a rigid sphere oscillating in an unbounded viscous liquid (see, e.g., ref (13)). One may expect such an approximation to hold for a relatively large (i.e., with respect to the penetration depth δ) particle as most of its surface is in contact with otherwise quiescent fluid located above the viscous penetration layer. Such assumption, however, requires justification since the unsteady viscous flow in a wall-bounded domain could be quite different from the unbounded flow.^23^ Obviously, for a particle of the size comparable to or smaller than the viscous penetration depth, this approximation does not apply. Moreover, the above approximation implicitly assumed that the hydrodynamic contribution to the contact force dominates over its fluid-mediated counterpart, which was entirely neglected.

The theory of hydrodynamic contribution to the QCM-D impedance due to adsorbed particles was recently developed in ref (24). This approach involved a number of ad hoc approximations and simplifying assumptions and was later revisited and extended in ref (25), where the excess shear force (or impedance) due to the presence of either freely suspended or rigidly attached (i.e., oscillating with a resonator as a while) discrete particles was determined analytically using a distant-particle asymptotic approximation. The closed-form expressions for the impedance and the velocity (linear and angular) of the freely suspended particle derived in ref (25) are in a very close agreement with the numerical (FEM) computations down to a rather close proximity of less than a particle radius. It was found, in particular, that for some realistic experimental conditions, the flow disturbance due to a layer of freely suspended (untethered) small particles located above the resonator produces the common (“inertial loading”) response with Δ*f* < 0 and ΔΓ > 0 of a magnitude of a few Hertz (at  = 5 MHz). However, Δ*f* can flip sign to positive depending on the value of *a*/δ and the proximity to the resonator. The same layer of adsorbed particles, however, results in the positive frequency shift and unorthodox negative bandwidth shift of some hundreds of Hertz. Notice the positive frequency shift, which is commonly associated with nonhydrodynamic effects, such as viscoelasticity of the adhesive contact (“elastic loading”), while ΔΓ < 0, implies reduced dissipation. The reason for the seemingly unphysical (sign- and magnitude-wise) response is that the analysis only concerned the excess shear due to the flow disturbance, whereas an adsorbed particle oscillating with a resonator as a whole excludes a fluid volume above it and also shields the resonator from the transverse shear wave that persists in the particle absence. The net excess shear force due to adsorbed particles should, however, combine the fluid-mediated force (as in ref (25)) and the contact force. In the present paper, we provide a detailed theoretical study of the net excess shear force (impedance) due to discrete adsorbates at low surface coverage in the analytically tractable limit of a stiff contact, which allows to decouple and analyze the role of hydrodynamics independently from other physical phenomena.

## Theoretical Section

### Problem Formulation

The viscous incompressible liquid in half-infinite space *z* > 0 is set into motion by the time-periodic horizontal oscillations of the infinite plane at *z* = 0 along the *x*-axis with frequency ω and amplitude *v*_0_ (see Figure 1). We further assume that a spherical particle of radius *a* firmly adheres to the plane and therefore oscillates with it in-sync without rotation. Assuming small amplitude of the oscillations, *v*_0_/ω ≪ *a*, to the leading approximation, the flow velocity ***V*** satisfies the unsteady Stokes equations
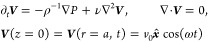
1where *P* is the pressure, ρ and ν = η/ρ are the density and the kinematic viscosity of the fluid, respectively, and the spherical distance *r* = |***x*** – ***x***_c_| is measured from the particle center located at ***x***_c_ = (0, 0, *h*). Although the particle adhesion corresponds to a vanishing separation distance, *h* ≈ *a*, we follow the general formulation^24,25^ and keep an arbitrary proximity *h* ≥ *a* in the analysis below. We introduce dimensionless variables by normalizing fluid velocity with *v*_0_, pressure with η*v*_0_/*a*, time with ω^–1^, and distance with *a*. Thus, the dimensionless (complex) flow field ***v*** and pressure *p* defined via ***V*** = *v*_0_ Re[e^–iω*t*^***v***] and *P* = η*v*_0_ Re[e^–iω*t*^*p*]/*a*, where Re stands for the real part, satisfy
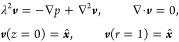
2Here, λ^2^ = −i*a*^2^ω/ν = −2i(*a*/δ)^2^. In the absence of a particle, the solution of eq 2 is given by , where λ = (1 – i)(*a*/δ) and *p*_0_ = 0.

When the particle is present, no analytical solution of eq 2 is readily available; however, some analytical progress is possible, e.g., for a distant particle (see ref (25)). The major aim of this paper is determining the *x*-component of the complex excess shear force (i.e., in excess to the shear force applied by the particle-free background flow), *F*, exerted on the oscillating plate in the incompressible viscous liquid due to an adsorbed particle.

For low values of the particle surface number density, *ñ*, when mutual hydrodynamic interactions between particles can be neglected, the dimensionless excess shear force *F*/η*av*_0_ due to a single particle translates into the dimensionless impedance,  probed by the QCM-D device. The net excess shear force *F* has two contributions: (i) the fluid-mediated contribution (screening or shielding force) due to the presence of the particle and (ii) the direct force the particle exerts on the surface via contact.

### Fluid-Mediated Force

The dimensionless stress tensor corresponding to {***v***, *p*} in eq 2 is defined by σ_*ik*_ = −*p*δ_*ik*_ + ∂_*k*_*v*_*i*_ + ∂_*i*_*v*_*k*_. In the absence of the particle, σ_*ik*_ has only *xz* and *zx* components, where at the plane *z* = 0 equals to −λ. If the particle is present, it modifies the stress exerted on the resonator by the fluid in the vicinity of the contact; however, far from the particle, we shall still have σ_*xz*_ ≈ −λ. Therefore, the net fluid-mediated excess shear force *F*_a_ (i.e., in excess of −λ times the surface of the resonator) exerted on the oscillating plate due to the presence of an adsorbed particle is defined by
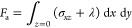
3The flow perturbation, , is governed by
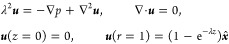
4The stress tensor σ_*ik*_^′^ = −*p*δ_*ik*_ + ∂_*k*_*u*_*i*_ + ∂_*i*_*u*_*k*_ corresponding to {***u***, *p*} in eq 4 obeys λ^2^*u*_*i*_ = ∂_*k*_σ_*ik*_^′^ and can be written via σ_*ik*_ as
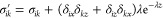
5Thus, *F*_a_ in eq 3 can then be written as
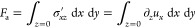
6

The direct numerical study of the force using eq 6 is problematic. The general structure of unsteady Stokes flows generated at the particle surface indicates that, at distances from the boundary greater than the viscous penetration depth δ/*a* ∝ |λ|^–1^, the flow ***u*** a is a superposition of a potential (inviscid) flow and an exponential correction.^13^ However, the dominant potential flow component makes no contribution to the viscous shear force in eq 6. Hence *F*_a_ is controlled entirely by the exponentially small correction to the potential flow. This renders accurate numerical computation of *F*_a_ over an infinite plate in eq 6 challenging.

We rewrite *F*_a_ in the form which is more suitable for the numerical study by using the Lorentz reciprocity.^26,27^ For an arbitrary incompressible dual flow satisfying , we have
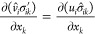
7Integrating eq 7 over the fluid volume in the semi-infinite domain, applying the divergence theorem, and using the original flow field ***v*** satisfying eq 2 as the dual flow, we find that
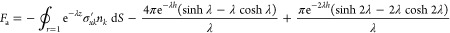
8where we made use of eq 5, giving the traction at the particle surface as σ_*xk*_*n*_*k*_ = −λe^–λ*z*^ cos θ + σ_*xk*_^′^*n*_*k*_, where θ is the polar spherical angle. Thus, instead of integration over the infinite plane at *z* = 0 in eq 6, the excess shear force *F*_a_ can be alternatively evaluated by integrating the traction σ_*xk*_^′^*n*_*k*_ over the particle surface at *r* = 1. Notice also that the last two (analytical) terms in the r.h.s. of eq 8 comprise (up to a factor of π) the net hydrodynamic contribution to the impedance due to the particle near contact reported in ref (24). The numerical results indicate that the first (integral) term is usually dominant over the last two (analytical) terms.

### Contact Force and Torque

For freely suspended particles, the excess shear force exerted on the resonator is mediated solely by the suspending fluid.^25^ The adsorbed particle not only modifies the flow above the resonator (i.e., via *F*_a_) but also applies a force to it via contact. We assume that the contact force the firmly attached particle exerts on the plane, *F*_c_, is equal in magnitude and opposite in sign to the force that the plane exerts on the particle (see also ref (21)). The contact force *F*_c_ is determined from the Newton’s force balance:
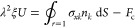
9where for a particle moving with a plane as a whole, its dimensionless translation velocity *U* = 1 and the traction σ_*ik*_*n*_*k*_ correspond to the original flow in eq 2. Here, the parameter ξ = *m*/ρ*a*^3^, where *m* stands for the particle’s mass, characterizes the solid inertia.

Substituting the traction at the particle surface σ_*xk*_*n*_*k*_ = −λe^–λ*z*^ cos θ + σ_*xk*_^′^*n*_*k*_ into eq 9 yields the following result:
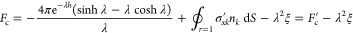
10where *F*_c_^′^ is the hydrodynamic part of the contact force. The net excess shear force due to an adsorbed particle can now be found as *F* = *F*_a_ + *F*_c_. Notice that upon neglecting the hydrodynamics entirely, the net excess force is due to inertial mass of the particle, *F* = −λ^2^ξ = −(4π/3)(ρ_s_/ρ)λ^2^ = i*m*ω/η*a*, being equivalent to the (dimensional) impedance . Substituting ω =  (where *n* is the odd overtone order) and using the small-load approximation for the above impedance, we readily arrive at the classical Sauerbrey equation:^1^
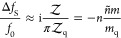
11where /(2*f*_0_) and *mñ* is the areal mass density (both have units of mass per unit area).

The contact torque *L*_c_ (the *y*-component, scaled with η*a*^2^*v*_0_) the adsorbed particle exerts on the resonator could also be of interest toward estimating the stiffness of the contact and it is given by (with respect to the particle center at *z* = *h*)
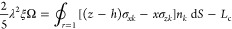
12where Ω is the dimensionless angular velocity of the particle scaled with *v*_0_/*a*. For an adsorbed particle with a stiff contact (i.e., without rotation, Ω = 0), there is no contribution of the solid inertia in the l.h.s. of eq 12 and the contact torque reduces to
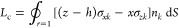
13The contact torque in eq 13 can be rewritten as an integral over the perturbed traction σ_*ik*_^′^*n*_*k*_ using eq 5 as (cf. eq 22 for  in ref (25))
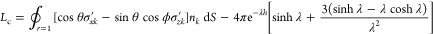
14If contact torque with respect to the point of contact (at *z* = 0) is considered, then we readily have
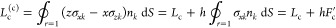
15where *F*_c_^′^ is the hydrodynamic part of the contact force in eq 10.

Notice that the above derivations of *F*_a_ and *F*_c_ are rigorous and do not involve any approximation, besides from the assumption of small-amplitude oscillations that allowed neglect of the nonlinear inertia terms in the flow equations. The resulting expressions involve integrals of the traction associated with the perturbed flow, σ_*ik*_^′^*n*_*k*_, over the particle surface at *r* = 1 that can be performed numerically.

### Small-Particle Limit

Let us consider the small-particle (or low-frequency) limit, |λ| ≪ 1, for which the steady Stokes equations hold to the first approximation as the unsteady term λ^2^***u*** in eq 4 produces  corrections in the solution.^28^ We next expand the perturbed flow ***u*** in eq 4 as ***u*** = λ***u***_1_ + λ^2^***u***_2_ + ..., *p* = λ*p*_1_ + λ^2^*p*_2_ + ..., and at the leading order we have
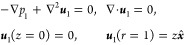
16Notice that the analytical terms in the r.h.s. of eqs 8 and 10 are all , meaning that at the leading order , we have
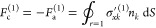
17In other words, for small particles, the fluid-mediated contribution is compensated by the (hydrodynamic part of) contact force to the leading approximation in λ, such that the net excess force due to an adsorbed particle *F* = *F*_a_ + *F*_c_ reduces to . Equation 16 governs the problem of a steady linear shear flow past a fixed sphere in contact with a plane wall, and its exact solution using special “tangent sphere” coordinates is given in ref (29). In particular, the dimensionless contact force to the leading approximation found from eq 17 is given by *F*_c_ = −*F*_a_ = −6π*f*λ + , where the constant *f* ≈ 1.7005.^29,30^

Analogously, at |λ| ≪ 1, the torque applied on the adsorbed particle can be estimated: the second (analytical) term in eq 14 is , and the integral term to the leading approximation contributes *L*_c_ ≈ −4π*g*λ, where the constant *g* ≈ 0.944.^30,31^ Given the asymptotic behavior of *F*_c_ above, we readily find that at contact (*h* = 1), the torque with respect to the point of contact to the leading approximation yields *L*_c_^(c)^ ≈ −(6*f* + 4*g*)πλ = −13.981πλ.

At the subleading order of , we have the following problem:
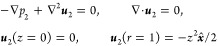
18The solution of eq 18 that would allow determining the subleading corrections to *F*_a_^(2)^ and *F*_c_^(2)^ is possible following the analysis in ref (29).

Moreover, using eqs 8 and 10, we find that due to the mutual cancelation of the terms involving σ_*xk*_^′(2)^ in *F*_a_^(2)^ and *F*_c_^(2)^, the net excess shear force to the leading approximation is yet determined by σ_*xk*_^′(1)^ in eq 16
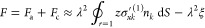
19where the surface integral can be evaluated using the revised solution of ref (29) giving the value of ≃−40.159.^31^ Thus, the impedance at the leading approximation is ∝ λ^2^ and determined by the interplay of the viscous and inertia forces; the ratio of the integral (viscous) term to the Sauerbrey (inertia) term in eq 19 is equal to ≃9.59 for neutrally buoyant particles. Notice that since λ^2^ = −i*a*^2^ω/ν is purely imaginary, the leading contribution to the real part of *F* reduces to , implying that for small particles, the dissipation shift is expected to be smaller in comparison to the frequency shift by a factor ∝ |λ| (see Figures 3c and 4a below).

## Numerical Computations

The numerical solution of eq 4 is performed in the dimensionless cylindrical coordinates {ϱ, ϕ, and *z*} (all distances scaled with *a*), such that *x* = ϱ cos ϕ and *y* = ϱ sin ϕ, with its origin at the plate at *z* = 0 and the *z*-axis passing through the center of the adsorbed spherical particle.

We use the following ansatz admitting simple dependence on the azimuthal angle:  =  =  =  and *p* =  which reduces the solution to two spatial dimensions.^25,32^ The corresponding problem for , , , and  is defined in the rectangular domain 0 ≤ ϱ ≤ ϱ_*m*_, 0 ≤ *z* ≤ *z*_*m*_ with an exclusion of the half unit disk centered at (0, *h*) representing the adsorbed particle. The pressure  is set to a fixed (zero) value far from the particle at *z* = *z*_max_ and ϱ = ϱ_max_. The boundary condition ***u*** = 0 is applied at ϱ = ϱ_max_, *z* = 0, and *z* = *z*_max_. We set no-flux boundary condition at ϱ = 0, while at the boundary of half-circle, we specify  and . We then apply the FEM implemented in Mathematica 12.0 to solve eq 4. A typical mesh size is selected to be 0.05 within the domain and 0.025 along the boundaries. Notice that for stiff contact, the particle is oscillating in-sync with the resonator and there is no relative shearing (or sliding) motion between the two. In ref (25), the fluid-mediated part of the excess shear force (*F*_a_) for an adsorbed particle was determined via the numerical solution of the auxiliary problem corresponding to a stationary (heavy inertial) particle located above the resonator, and this resulted in numerical difficulties at close proximity owing to strong lubrication forces. The direct formulation of the problem in eq 4 circumvents these complications, allowing for an accurate numerical solution near contact, *h* → 1.

**Figure 2:**
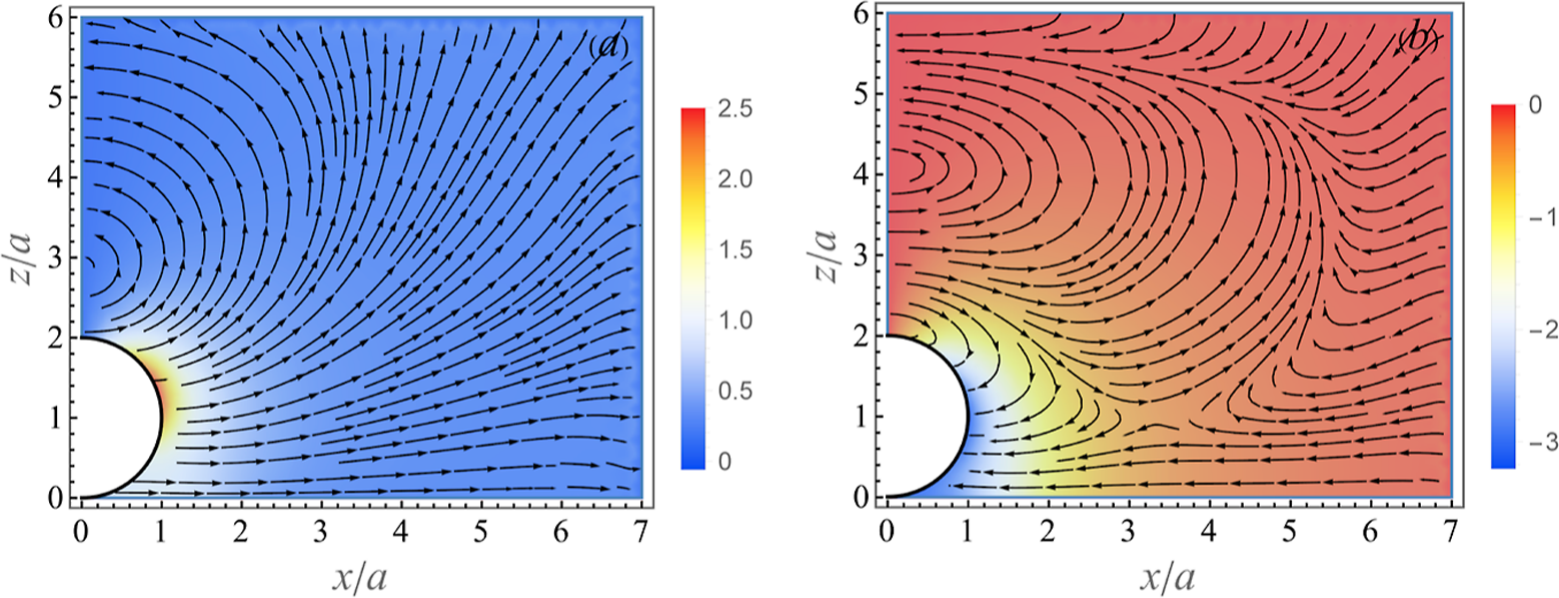
Perturbed flow (streamlines) and pressure (color map) fields in eq 2 due to an adsorbed particle for δ/*a* = 1 in *xz*-plane (for ϕ = 0) at two different time instances: (a) velocity  and pressure  corresponding to ω*t* = 0 and (b) velocity  and  corresponding to ω*t* = π/2.

Numerical computation shows that flow ***u*** converges at ϱ_max_ ≃ 9 and *z*_max_ ≃ 9 + *h*. The typical flow and pressure disturbance due to an adsorbed particle for δ = 1 and *h* = 1.001 in the meridional plane *xz*–plane (for ϕ = 0) are shown in Figure 2a,b at two instances, ω*t* = 0 and ω*t* = π/2, respectively. It can be readily seen that the interaction of the transverse wave originated at the oscillating plate (see the undisturbed velocity in Figure 1) with the particle creates a rather complex flow pattern with transient recirculations.

The predictions of the FEM computations are compared to the results of numerical simulations using the frequency-domain lattice Boltzmann method (FreqD-LBM) to solve the oscillatory Stokes equations. FreqD-LBM was first proposed in ref (33), and it amounts to a variant of the LBM, where the populations are replaced by complex amplitudes of the oscillation. FreqD-LBM maintains the simplicity of the conventional LBM and also covers viscoelasticity.^18^ A typical simulation box contains 20 × 20 × 20 nodes and typical times per run range between a few minutes and a few hours on a standard desktop computer. Following ref (19), the particle is not part of the simulation volume, while its surface is part of an oscillating boundary. Differing from ref (19), we assumed infinite contact stiffness. Bounce back of the populations from the surface causes a transfer of momentum to the sphere, which is transferred to the resonator through contact. The fluid-mediated force results from the momentum transfer during bounce back of the populations at the resonator surface. Periodic boundary conditions were applied at the side boundaries, while at the top boundary (located at the height of 2.5*a*), the populations were matched to an analytical solution. The accuracy of FreqD-LBM is constrained by the surface coverage (i.e., the finite lateral size of the simulation box) and the grid resolution, Δ*x* ≪ δ; insufficient grid resolution results in fictitious fluid viscoelasticity. Therefore, the simulations were performed using Δ*x*/δ < 0.03 for particles with *a*/δ ≲ 0.4 (see Figure 4a,b below). FreqD-LBM simulations for larger adsorbates would require considerably longer CPU times, the limitation which could potentially be resolved by parallelizing the code. This undertaking is beyond the scope of the present paper and will be reported elsewhere.

## Experimental Section

### Materials

All chemical reagents comprising sodium chloride, sodium hydroxide, hydrochloric, and sulfuric acids were commercial products of Sigma-Aldrich and were used without additional purification. Ultrapure water was obtained using the Milli-Q Elix&Simplicity 185 purification system from Millipore.

Positively charged (amidine) and negatively charged (sulfonate) polymer particles supplied by Invitrogen (Life Technologies Polska Sp.z.o.o., Warsaw, Poland) were used in the deposition kinetic measurements carried out by QCM.

The gold/quartz/silicon dioxide (SiO_2_) sensors were supplied by Q-Sense, Gothenburg, Sweden, whereas the bare gold sensors were supplied by QuartzPro, Jarfalla, Sweden. Both sensor types were characterized by a fundamental frequency of 5 MHz. Before each measurement, the sensors were cleaned in a mixture of 96% sulfuric acid (H_2_SO_4_), hydrogen peroxide (30%), and ultrapure water in volume ratio of 1:1:1 for 10 min. Afterward, the sensor was rinsed by deionized water at 80 °C for 30 min and dried out in a stream of nitrogen gas.

### Methods

The bulk concentration of particles in the stock suspension was determined by a dry mass method. Before each experiment, the stock suspension was diluted to the desired concentration by pure NaCl solutions with the pH adjusted to either 4 (by adding HCl) or 5.6 (by adding pure distilled water). The particle density was determined by the densitometry/dilution method. The diffusion coefficient of the particles was determined by dynamic light scattering using the Zetasizer Nano ZS instrument from Malvern. The hydrodynamic diameter was calculated using the Stokes–Einstein relationship. The particle size was determined independently by atomic force microscopy (AFM). The electrophoretic mobility of the particles was acquired using laser Doppler velocimetry using the same apparatus. The particles’ zeta potential was calculated using the Henry formula.

Relevant parameters characterizing the topography of the sensors comprising the average height, root-mean-square (rms), the skewness, and the roughness correlation length were determined by AFM imaging, carried out under ambient conditions in a semicontact mode.^34^

In the case of the positively charged amidine particles, the QCM measurements were carried out according to the standard method described in ref (35) using the Q-Sense Instrument. First, a stable baseline in pure electrolyte of a fixed concentration was attained in the QCM-D cell (Q-Sense window cell QWM401) under a controlled volumetric flow rate. Afterward, the particle suspension of a fixed concentration was flushed at the same flow rate. Finally, the desorption run was initiated, where a pure electrolyte solution of the same pH and ionic strength was flushed through the cell. In the case of the negatively charged sulfonate particles, a macrocation (poly allyl chloride, PAH) adsorption step was first performed before initiating the particle deposition run.

The real particle mass coverage at the sensor was determined using the AFM method, as previously described.^35^ Accordingly, a QCM run was stopped after completing the desorption step, and the sensors were removed from the suspension, carefully dried under a controlled humidity, and was imaged by AFM under ambient air conditions. The particle surface number density was determined by a direct counting of over a few equally sized areas randomly chosen over the sensor with a total number of particles of about 1000. The AFM particle areal mass density was calculated as mñ, where *m* is the single particle mass.

Particle deposition kinetics in the QCM cell was theoretically evaluated in terms of the hybrid convective-diffusion and random sequential adsorption approach using as the AFM-derived real particle coverage as a control.^35^

Further details of the experimental procedures, the relevant physicochemical characteristics of the particles, and the theoretical modeling are provided in the Supporting Information.

## Results and Discussion

**Figure 3:**
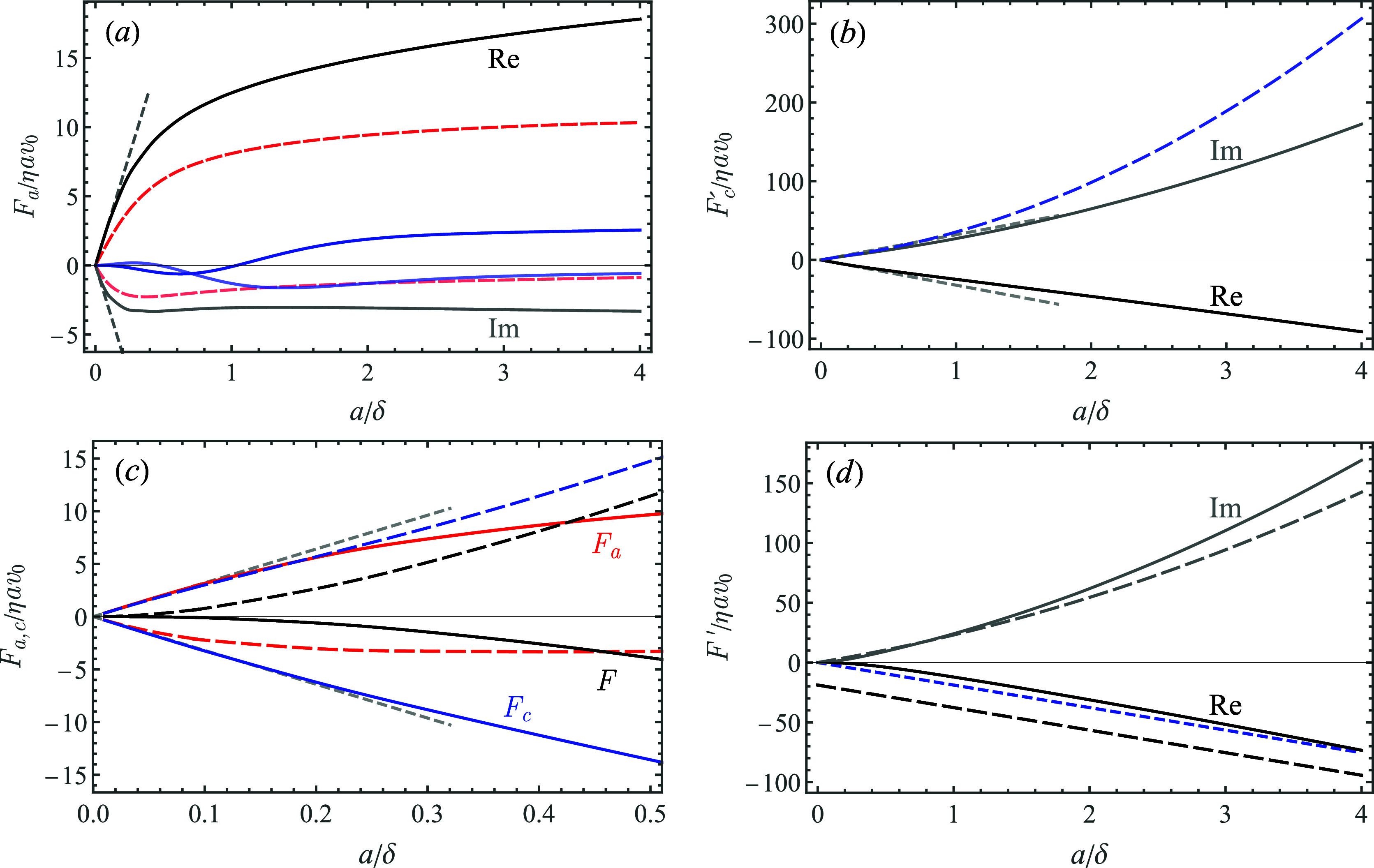
Excess shear force (real and imaginary part) due to adsorbed particles (*h* = *a*) vs *a*/δ. (a) Fluid-mediated contribution *F*_a_: the solid (black) lines stand for the numerical results, short-dashed (gray) lines for the small-λ asymptote, *F*_a_^(1)^ and long-dashed (red) lines correspond to the distant-particle prediction *F*_a_^asym^ at *h* = *a* in eq 20; and the blue curves stand to the analytic part (last two terms) of *F*_a_ in eq 8; (b) hydrodynamic part of the contact force *F*_c_^′^ (black, gray); the short dashed lines for the small-λ asymptote *F*_c_^(1)^ and long-dashed (blue) line for the imaginary part of the net contact force *F*_c_ (the real part is unchanged) for neutrally buoyant particles (for ξ = 4π/3); and (c) various components of the excess force for *a*/δ ≲ 0.5: *F*_c_ (blue, for ξ = 4π/3), *F*_a_ (red), and *F* (black); solid and long-dashed lines stand for real and imaginary parts of different terms, respectively. (d) Comparison of the net excess force *F*′ (excluding solid inertia, solid lines) vs the analytical result *F*_0_ for a sphere oscillating in an unbounded liquid^13^ (long-dashed lines); short-dashed (blue) curve stands for the real part of *F*_0_ upon subtracting the pseudo-Stokes drag term (−6π) in eq 21.

The numerical (FEM) results for the real and imaginary parts of the excess shear force due to an adsorbed particle at contact (*h* = *a*) are presented in Figure 3a–d (solid curves). The fluid-mediated contribution *F*_a_ in eq 8 is depicted in Figure 3a vs *a*/δ together with the linear small-λ asymptotes *F*_a_^(1)^ (short-dashed lines) and the prediction of the distant-particle theory (long-dashed, red curves) that assumes *h* ≫ max(*a*, δ), while the ratio *a*/δ is not constrained^25^
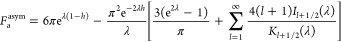
20Here, *I*_ν_(λ) and *K*_ν_(λ) are the modified Bessel functions of the first and second kind, respectively. It can be readily seen that the numerical results show excellent agreement with *F*_a_^(1)^ at low values of *a*/δ. The agreement with the theoretical prediction in eq 20 is only qualitative. Recall that starting from relatively small separations, *h* ≳ 1.5*a*, a surprisingly close agreement between the numerical results and eq 20 was found,^25^ while at contact (*h* = *a*), the theory considerably underestimates the fluid-mediated contribution, *F*_a_ (i.e., both the real and the imaginary parts; see red long-dashed curves in Figure 3a). Another observation is that the relative weight of the analytical (the last two) terms in eq 8 to *F*_a_ is small for all values of *a*/δ (see the blue curves in Figure 3a).

Notice that Re[*F*_a_] > 0, while Im[*F*_a_] < 0, which implies positive frequency shift (which is typically associated with nonhydrodynamic effects, such as contact viscoelasticity), and ΔΓ < 0, indicating reduced dissipation. The reason for seemingly unorthodox result is that the adsorbed particle excludes a fluid volume above the resonator and at the same time shields the resonator from the shear wave that would otherwise persist in its absence. One might expect that adding the contact force would flip the signs of the net excess force (see below).

The numerical results for the hydrodynamic part of the contact force (i.e., excluding solid inertia), *F*_c_^′^ (the sum of the first two terms in eq 10), are depicted in Figure 3b vs *a*/δ (solid curves). The linear small-λ asymptotes *F*_c_^(1)^ (short-dashed lines) approximate *F*_c_^′^ very well up to *a*/δ ≈ 1. The long-dashed (blue) line stands for the net contact force *F*_c_ in eq 10 for a neutrally buoyant particle with ξ = 4π/3. It can be readily seen that for *a* ≳ δ, the excess force is dominated by the contact force, as *F*_c_ ≫ *F*_a_, while for *a*/δ ≲ 0.5, the two terms are comparable. Moreover, since *F*_a_^(1)^ = −*F*_c_^(1)^, their contributions compensate each other and the net effect is . This notion is illustrated in Figure 3c, where we plot *F*_a_, *F*_c_ (for neutrally buoyant particle, ξ = 4π/3) and the resulting net excess force *F* vs *a*/δ < 0.5. The small-λ linear asymptotes are shown as short dashed lines. The exact cancelation of the fluid-mediated and contact forces at the leading order in λ result in rather low values of *F* for small particles, in particular its real part of , while the imaginary part is of  (see the analysis above). For example, for 50 nm (*a*/δ = 0.1) neutrally buoyant particles in water for the fundamental frequency of  = ω/2π = 5 MHz, giving δ ≈ 252 nm, yields  .≈  Using the small-load approximation,^2^ the shift in oscillation frequency, Δ*f*, and in its half-bandwidth, ΔΓ (or the dissipation factor,  = ), can be found from  = , where the quartz resonator’s shear wave impedance  kg/m^2^s and the oscillation frequency  where *n* is the (odd) overtone number. Assuming the particle number density at the surface of the resonator  (i.e., one nanoparticle per 100*a*^2^ surface area), the small-load approximation at the fundamental frequency  yields Δ*f* ≈ −56.0 Hz and ΔΓ ≈ 7.4 Hz.

In Figure 3d, we compare the hydrodynamic part of the net excess shear force, *F*′ (excluding the solid inertia term, solid curves) vs the classical result for the force exerted on an rigid sphere oscillating with velocity  in an unbounded viscous liquid, quiescent at infinity (long-dashed lines). This force can be written in the dimensionless form (scaled with η*av*_0_) as (see ref (13))
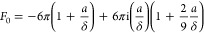
21

It was previously proposed,^22^ that for large enough particles (*a* ≫ δ), the hydrodynamic contribution to the impedance can be closely approximated by *F*_0_ as most of the particle surface oscillates in almost quiescent liquid located above the penetration depth δ. It can be seen that the agreement between numerical result for Im[*F*′] (dashed line) and the second (“added mass”) term in eq 21 is quite close and the relative error (which increases with *a*/δ) is ∼16% for *a*/δ = 4. For the same value of *a*/δ, the real part, Re[*F*′], deviates from the first (“drag”) term in eq 21 by ∼22%, while this error becomes larger for smaller particles: it is already ∼68% for *a*/δ = 1. It appears that subtracting the zero-frequency pseudo-Stokes drag term −6π from Re[*F*_0_] yields much closer agreement (see the short-dashed line in Figure 3d). The rationale behind subtraction of the zero-frequency (pseudo-Stokes) drag term is as follows. In the small-particle (or, alternatively, low-frequency) limit, *a* ≪ δ, the flow disturbance is equivalent to that of the wall-bounded shear flow, and the net contribution of the hydrodynamics to the excess shear force is small, ∼(*a*/δ)^2^. The pseudo-Stokes term corresponds to a zero-frequency limit of the force exerted on a particle oscillating in an unbounded fluid, and it is . Thus, clearly the pseudo-Stokes term is irrelevant for small adsorbates. In the opposite limit, *a* ≫ δ, the pseudo-Stokes term is small in comparison to other terms in eq 21, and its omission should not significantly alter the result. Apparently, subtracting the pseudo-Stokes term in eq 21 greatly improves the accuracy of the ad hoc model^22^ all across the scale of *a*/δ.

**Figure 4:**
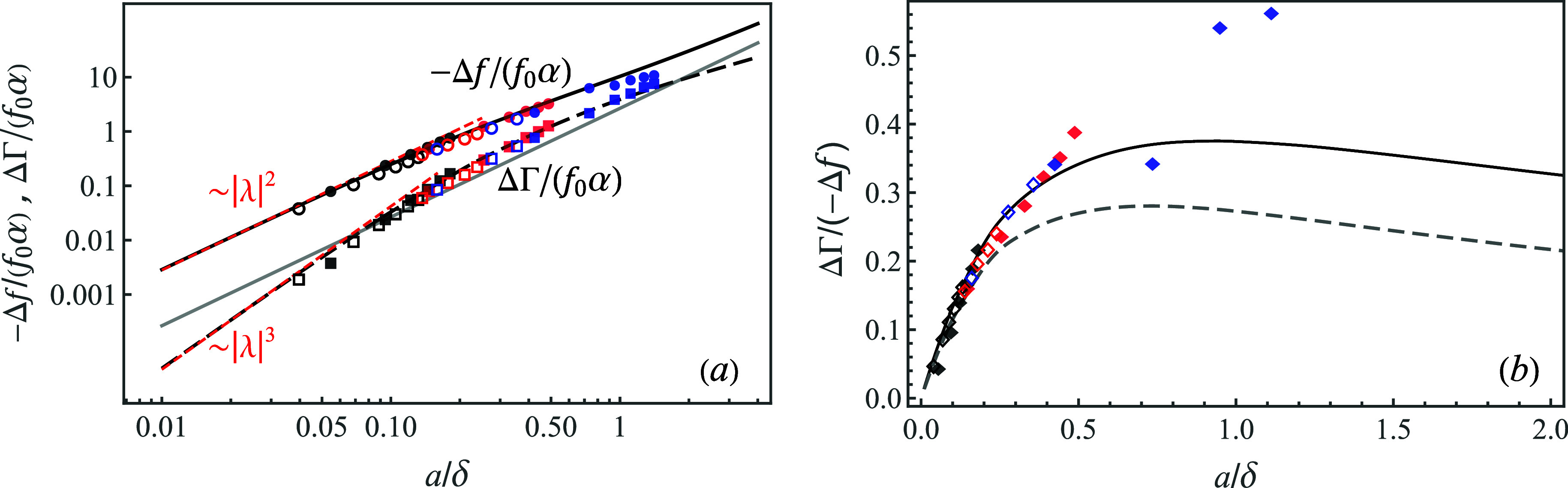
(a) Dimensionless frequency shift  (solid black curve) and the half-bandwidth  (long-dashed curve) shift due to adsorbed neutrally buoyant (ρ_s_/ρ = 1) adsorbates vs *a*/δ (log–log plot); the short-dashed (red) lines show the asymptotic behavior at *a*/δ ≪ 1; and the solid (gray) line is the Sauerbrey frequency shift,  for comparison. Empty symbols (circles and squares) are the results of FreqD-LBM simulations for 20 nm (black), 40 nm (red), and 80 nm (blue) particles. Full symbols are the experimental results for 26 nm (black) and 73 nm (red) amidine polystyrene particles adsorbing onto the SiO_2_ sensor and 205 nm sulfonate polystyrene particles (blue) adsorbing onto the Au sensor. Different values of *a*/δ for the same color correspond to different overtones, *n* = 1–11. (b) Acoustic ratio ΔΓ/(−Δ*f*) vs *a*/δ for neutrally buoyant particles (solid line); full symbols are the experimental results and empty symbols stand for the FreqD-LBM simulation results; and the dashed line is the theoretical prediction for heavy particles with ρ_s_/ρ = 2.44 shown for comparison.

The dimensionless frequency shift,  and the half bandwidth shift,  vs *a*/δ, for neutrally buoyant particles (i.e., ρ_s_/ρ = 1) are shown as double-log plot in Figure 4a (the black solid and dashed curves, respectively). Here,  is the dimensionless (viscous-to-solid) impedance ratio. For example, for 50 nm diameter particles in water and particle areal density , we find that α = 4.55 × 10^–5^. Both shifts are monotonically increasing functions of *a*/δ, while for small particles, eq 19 yields
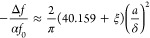
22which for neutrally buoyant particles (ξ = 4π/3) gives  ≈ 28.23(*a*/δ)^2^ (see the short-dashed line in Figure 4a). Note that for small adsorbates, −Δ*f* ∝ (*a*/δ)^2^ ∼ ω and ΔΓ ∝ (*a*/δ)^3^ ∼ ω^3/2^. At higher values of *a*/δ ≳ 0.2, the crossover to a sublinear dependence Δ*f* ∼ ω^0.82^ takes place, and similarly, the crossover to ΔΓ ∼ ω^0.64^ occurs for *a*/δ ≳ 0.5. As a result, the acoustic ratio, ΔΓ/(−Δ*f*), shows a nonmonotonic dependence on *a*/δ, reaching the maximum value at *a*/δ ≈ 0.9 (see Figure 4b).

The theoretical results are in excellent agreement with the experimental results (full color symbols) and the results of direct numerical simulations by FreqD-LBM (empty symbols) over a wide range of particle sizes and frequencies. Notice that for *a*/δ > 1, the stiff contact between larger 205 nm adsorbates (blue symbols) and the resonator cannot be formed due to high particle inertia and elevated viscous component of the contact force, resulting in substantial deviation from the theory. The scaled frequency shift due to the solid inertia alone by the Sauerbrey eq 11,  = (8/3)(ρ_s_/ρ)(*a*/δ)^2^, for neutrally buoyant particles is depicted for comparison (solid gray line). It can be readily seen that this equation significantly underestimates the mass of discrete adsorbates in liquids. In particular, in the small-particle limit, *a*/δ → 0, we found that Δ*f*/Δ*f*_S_ ≈ 10.8, rendering the standard Sauerbrey model highly inaccurate.

The theoretical prediction of the dimensionless acoustic ratio, ΔΓ/(−Δ*f*), being independent of the surface coverage *ñ* is depicted vs *a*/δ for neutrally buoyant particles in Figure 4b (solid line) together with the experimental data (full diamonds, the same as in Figure 4a) and FreqD-LBM simulations results (empty diamonds). There is a close agreement between the theoretical prediction, experiment, and simulations. For larger 205 nm particles, the agreement can only be observed at low overtones (*n* = 1, 3) due to the greater effect of the inertia and the viscous force acting on the particle, as explained above. Notice that for adsorbed particles with stiff contact, the theory predicts that the acoustic ratio has a maximum; e.g., for neutrally buoyant particles, it is ΔΓ/(−Δ*f*) ≈ 0.38 at *a*/δ ≈ 0.9. Heavier particles are expected to yield lower values of the acoustic ratio at the maximum as the inertial (Sauerbrey) term −λ^2^ξ in eq 10 contributes solely to the imaginary part of *F*, increasing the (negative) frequency shift, (−Δ*f*), while ΔΓ remains unchanged. For instance, for silica nanoparticles suspended in ethanol^36^ (ρ_s_/ρ = 2.44, dashed line in Figure 4b), we find ΔΓ/(−Δ*f*) ≈ 0.28 at *a*/δ ≈ 0.7.

**Figure 5:**
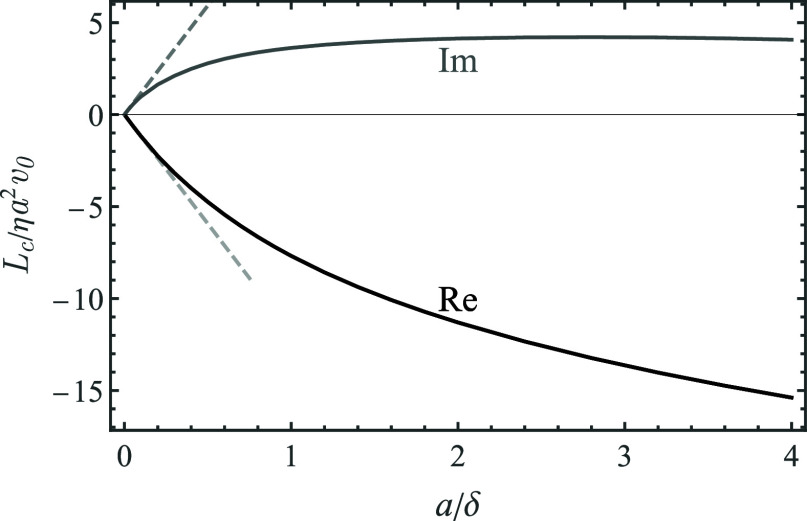
Contact torque *L*_c_/η*a*^2^*v*_0_ with respect to the particle center vs *a*/δ. Solid (black) and long-dashed (gray) curves stand for the numerical results for the real and the imaginary parts of *L*_c_; the short-dashed (gray) lines stands for the small-λ asymptotics.

Finally, the real and imaginary parts of the contact torque *L*_c_/η*a*^2^*v*_0_ (with respect to the particle center at *z* = *h* in eq 14) are depicted vs *a*/δ in Figure 5. The small-λ asymptotic (short-dashed lines) shows excellent agreement with the numerical results (black solid and gray long-dashed curves).

Notice that the torque *L*_c_^(c)^ with respect to the point of contact in eq 15 would be much higher owing to the large contact force, as  The viscous torque exerted on the discrete adsorbates is relevant toward the prospective investigation of the competition between hydrodynamic and adhesive forces controlling the contact dynamics in a more general formulation, where a priori assumption of the stiff contact is relaxed.

## Conclusions and Perspectives

In the present paper, we dissect the role of hydrodynamics in the QCM-D frequency response due to discrete adsorbates in liquids. Under the assumption of low surface coverage, we theoretically study the excess shear force (or the complex impedance) exerted on the resonator for the analytically tractable case of a stiff contact between a single adsorbed rigid particle and the resonator. Using the reciprocal theorem for unsteady Stokes flows, we consider two separate contributions to the impedance: (i) due to the flow disturbance by the particle and (ii) due to the viscous force exerted on the particle and transmitted to the sensor via contact. The contribution (i) is fluid-mediated and does not require physical contact. For an arbitrary particle size or frequency (i.e., arbitrary value of *a*/δ), the resulting hydrodynamic problem is solved numerically, while in the small-particle limit, *a*/δ ≪ 1, we construct the solution as an asymptotic expansion in powers of λ = (1 – i)(*a*/δ). The asymptotic solution shows that for small particles, *a*/δ ≪ 1, the contribution (i) to the impedance at the leading (linear) approximation in λ is compensated exactly by the contribution (ii), such that the net impact of the hydrodynamics to the impedance reduces to . Therefore, for small values of *a*/δ, the resulting frequency shift is shown to scale as −Δ*f* ∼ (*a*/δ)^2^ ∝ ω and half-bandwidth shift, ΔΓ ∼ (*a*/δ)^3^ ∝ ω^3/2^. The theoretical predictions for the frequency and half-bandwidth shifts and the acoustic ratio, ΔΓ/(−Δ*f*), show excellent agreement with the experimental results and the numerical (FreqD-LBM) simulations in a wide range of particle sizes and frequencies. Our theoretical results can be applied for precise analysis of the QCM-D kinetic data, providing essential information, such as the adhesion strength, on bioparticle interaction with abiotic interfaces in liquids.

It is demonstrated that for small particles, the hydrodynamic forces dominate over the inertial forces, and, as a result, commonly used Sauerbrey eq 11, which evaluates the frequency shift Δ*f*_S_ based solely on the inertia of the adsorbates (i.e., entirely neglecting the hydrodynamics), significantly underestimates their mass. In particular, we found that for small neutrally buoyant adsorbates, the apparent mass derived from QCM-D, independent of the overtone, is about 10 times the Sauerbrey (inertial) mass, as Δ*f*/Δ*f*_S_ ≈ 10.8 in the limit *a*/δ ≪ 1. We would also like to emphasis that the early hypothesis of the “trapped liquid” (e.g., refs (10–12)) is fundamentally wrong as the source of discrepancy is not of a static, but of a hydrodynamic origin, i.e., due to nontrivial flow disturbance caused by the adsorbate.

Let us briefly address the assumptions used in the present study. The revised analysis of ref (29) demonstrates^30^ that in the limit *a* ≪ δ, the mutual hydrodynamic interaction between discrete adsorbates is negligible for the surface coverage . Ref (21) reports on θ ≲ 5% as the low-coverage threshold based on the results of IBM simulations, while the FreqD-LBM simulations used in this work suggest a slightly lower limit of θ ≲ 3%. All of these estimates are roughly in accord with each other.

Throughout the paper, we implicitly applied the kinematic condition of a stiff contact (no “sliding” or “rocking”), while in practice, the contact stiffness is determined by the competition between adhesion, viscous, and inertial forces. It can be shown (see the Supporting Information for details) that the amplitude of the particle displacement with respect to the resonator scales as *a*^2^ and ω^3/2^, which is in a qualitative agreement with the experimental findings, showing the increasing deviation from the theory upon increasing the particle size or oscillation frequency (see Figure 4a,b).

We have previously shown that in agreement with the argument in ref (24), in the limit of vanishing proximity, ϵ = *h*/*a* – 1 → 0, the translation and rotation velocities of a freely suspended spherical particle to the leading approximation in ϵ tend to these of a rigidly attached particle, i.e., *V* – 1, Ω ∼ | ln ϵ|^–1^ entirely due to the strong lubrication forces (see Section V in ref (25)). However, the fluid-mediated contribution to the excess shear force due to a freely suspended particle near contact, that can be written as , might be different from the corresponding contribution due to an adsorbed particle, *F*_f_ ≠ *F*_a_, due to logarithmic dependence of the corresponding resistance functions,  and  at ϵ → 0. The analysis of the QCM reading due to attachment of the particle is beyond the scope of the present paper and will be considered elsewhere.

The quantitative interpretation of the QCM readings in liquids is complex, and low-surface coverage of spherical adsorbates provides the first sensible approximation. While our numerical (FEM) solution relying on the axial symmetry of the adsorbates could be readily extended to spheroidal particles and spherical cavities and caps, the analytical small-particle approximation only applies to spherical adsorbates. On the other hand, the numerical FreqD-LBM simulations can be used to simulate impedance due to discrete adsorbates of an arbitrary shape and surface coverage.

The idea of using QCM-D as a tool for quantifying viscoelastic properties of the soft contact between discrete adsorbates and the functionalized surfaces sounds appealing, but its implementation could be rather complex. The accurate account of hydrodynamics in such a case is critical as subtle differences in particles’ displacement produce large differences in the impedance, e.g., notice the difference in impedance due to freely suspended particles near contact (see refs (21 and 25)) and adsorbed particles analyzed here. The soft compliant contact allows particle motion relative to the resonator (rocking or sliding^14^) which is controlled by the competition between (a priori unknown) adhesive and hydrodynamic forces, rendering the quantitative analysis of the QCM-D signal complicated.

In experiments, increasing the size of the discrete adsorbates and/or oscillation frequency prevent formation of the stiff adhesive contact due to the augmented effect of the solid inertia and viscous drag force, suggesting that perhaps accurate gravimetric measurements of large discrete adsorbates are possible at lower frequencies. Since the fundamental frequency of AT cut quartz is inversely proportional to its thickness, it is theoretically possible to build a device with a thicker crystal operating at lower resonant frequency.

## Supporting Information Available

Materials and methods, particle and QCM sensor characteristics, experimental determination of the impedance, theoretical predictions for the impedance and the acoustic ratio, and estimate of the QCM contact stiffness (PDF)
