# Supplementary material for: Quartz Crystal Microbalance Frequency Response to Discrete Adsorbates in Liquids
Source: Anal Chem. 2024 Jun 21;96(26):10559–68. doi: 10.1021/acs.analchem.4c00968 (PMC11223097; doi:10.1021/acs.analchem.4c00968)
Supplement: Supplementary file 1 — ac4c00968_si_001.pdf [file ac4c00968_si_001.pdf]

SUPPORTING INFORMATION

Quartz Crystal Microbalance frequency response to discrete adsorbates in liquids

Alexander M. Leshansky[†], Boris Y. Rubinstein[‡], Itzhak Fouxon[†], Diethelm Johannsmann[¶], Marta Sadowska[§], Zbigniew Adamczyk[§]

[†] Department of Chemical Engineering, Technion – IIT, Haifa 32000, Israel
[‡]Stowers Institute for Medical Research, 1000 E 50th st., Kansas City, MO 64110, USA
[¶]Institute of Physical Chemistry, Clausthal Universtity of Technology, Arnold-Sommerfeld-Straße 4, 38678 Clausthal-Zellerfeld, Germany
[§]Jerzy Haber Institute of Catalysis and Surface Chemistry, Polish Academy of Sciences, Niezapominajek 8, 30 - 239 Krakow, Poland;

e-mails:
lisha@technion.ac.il
bru@stowers.org
itzhak8@gmail.com
johannsmann@pc.tu-clausthal.de
marta.sadowska@ikifp.edu.pl
zbigniew.adamczyk@ikifp.edu.pl

Table of contents:

1. **Materials and Methods**
2. **Particle and QCM Sensor Characteristics**
3. **Experimental Determination of the Impedance**
4. **Theoretical Predictions for the Impedance and the Acoustic Ratio**
5. **Estimate of the QCM Contact Stiffness**

1. Materials and Methods

Materials. Positively and negatively charged polystyrene particles supplied by Invitrogen (Life Technologies Polska Sp.z.o.o., Warsaw, Poland) were used in the deposition kinetics measurements carried out by QCM.

The gold/quartz/silicon dioxide (SiO_2) sensors were supplied by Q-Sense, Gothenburg, Sweden. The bare gold sensors used in experiments were supplied by QuartzPro, Jarfalla, Sweden. Both sensor types were characterized by the fundamental frequency of 5 MHz.

Before every measurement, the sensors were cleaned in a mixture of 96 % sulfuric acid (H_2SO_4), hydrogen peroxide (30 %) and ultrapure water in volume ratio 1:1:1 for 10 minutes. Afterward, the sensor was rinsed by deionized water at 80° C for 30 min and dried out in a stream of a nitrogen gas.

Relevant parameters characterizing the topography of the sensors comprising the root mean square (rms), the surface height, the skewness, the roughness correlation length were determined by atomic force microscopy (AFM) imaging carried out under ambient conditions in a semi-contact mode.

Methods. The bulk concentration of particles in the stock suspension was determined by the dry mass method. Before each deposition experiment, the stock suspension was diluted to the desired concentration by pure NaCl solutions with the pH adjusted to either 4 (by HCl addition) or 5.6 (pure distilled water).

The diffusion coefficient of the particles was determined by the dynamic light scattering (DLS) using the Zetasizer Nano ZS instrument from Malvern. The hydrodynamic diameter was calculated using the Stokes-Einstein relationship. The electrophoretic mobility of particles was measured by the Laser Doppler velocimetry (LDV) technique using the same apparatus.

In the case of the positively charged amidine particles, the QCM measurements were carried out according to the standard procedure described in Ref. [1] using the Q-Sense window cell type QNM401 (Biolin Scientific, Stockholm, Sweden). Firstly, a stable baseline in pure electrolyte of a fixed concentration was attained in the QCM-D cell for defined flow rate (typically 2.5×10^{-3} cm^3 s^{-1}). Afterward, the particle suspension of a fixed concentration was flushed at the same flow rate. Finally, the desorption run was initiated where pure electrolyte solution of the same pH and ionic strength was flushed through the cell. In the case of the negatively charged sulfonate particles a macrocation (poly-allyl chloride, PAH), adsorption step was first performed before initiating the particle deposition run.

The geometry of the QCM cell is such that an oblique impinging 3D jet flow is formed at the inlet, providing quasi-uniform transport condition over the sensor surface. However, the flow distribution, which obviously varies between cells and cannot be predicted *a priori*, has a minimal impact on experimentally determined impedance components because the particle coverage was directly determined with AFM as an average from the entire sensor surface.

The deposition kinetics of particles was determined using the AFM method as previously described in Ref. [1,2]. Accordingly, a QCM run was stopped after completing the desorption step, the sensors was removed from the suspension, carefully dried under a controlled humidity and imaged under ambient conditions using the NT-MDT Solver BIO device with the SMENA SFC050L scanning head. The particle surface number density (i.e., the number of particles per a unit area, typically one square micrometer) denoted hereafter by N, was determined by a direct counting of over a few equal sized areas randomly chosen over the sensor with the total number of particles about 1000. Afterward, the particle mass coverage was calculated as $M = \tilde{n}m$, where m is the single particle mass.

The zeta potential of bare and PAH covered substrates was determined via streaming potential measurements performed according to the procedure described in Ref. [3] applying the Smoluchowski formula where the correction for the surface conductivity was considered.

All experiments have been performed at the temperature of 298 K, in 1 or 10 mM NaCl solutions.

2. Particle and QCM Sensor Characteristics

The particle density ρ_s determined by the densitometry/dilution method was equal to 1.05 g cm^{-3} for both the amidine and the sulfonate type. Their sizes and zeta potentials determined by DLS, AFM and LDV methods, respectively are given in Table S1.

Table S1. Basic physicochemical parameters of the polymer particles used in this work.

Particles[‡]	d_p [nm] DLS	d_p [nm] AFM	ζ [mV] LDV
A26 10 mM NaCl	25±3	26±3	71±2
A70 10 mM NaCl	69±5	73±5	74±2
L200 1 mM NaCl	200±10	205±10	-64 ±4

The surface topography of the sensors used in the QCM deposition kinetic measurements was determined by AFM imaging carried out under ambient air conditions[1,2]. Figure S1 depicts the images of the gold and the gold/silica sensors.

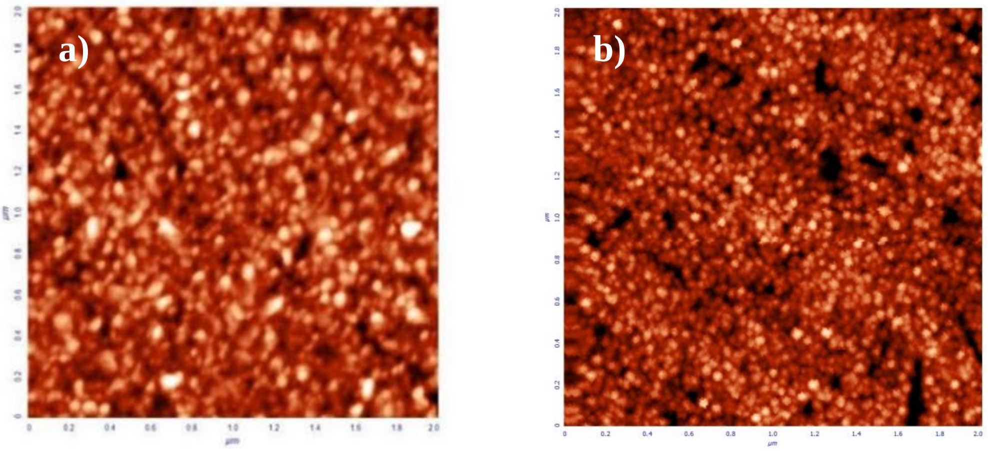

Figure S1. The AFM images of the sensors surfaces used in this work: a) the gold sensor; b) the gold/silica sensor.

[‡] A26 and A70 are the positively charged amidine particles, L200 are the sulfonate polystyrene particles; $d_p = 2a$ is the particle diameter; ζ is the zeta potential. DLS - dynamic light scattering, AFM - atomic force microscopy, LDV - Laser Doppler Velocimetry.

The most relevant parameters comprising the root mean square (rms), the surface height, the skewness characterizing the asymmetry of the height distribution and the roughness correlation length characterizing their lateral dimensions, are collected in Table S2.

Table S2. Basic topographical parameters of the bare gold and the gold/silica sensors derived from the AFM measurements.

Sensor	rms [nm]	Surface height [nm]	Skewness	Roughness correlation length [nm]
Bare gold	1.4±0.1	4.0±0.3	0.30±0.05	50±5
Gold/Silica	1.0±0.1	3.0±0.3	0.35±0.05	60±5

In general, the sensor topography, not only the rms factor, but also the surface height and the roughness correlation length, can play a significant role, particularly for larger particles. As can be readily seen from Table S2, the roughness correlation length was considerably larger than its average height. This suggests that its average radius of curvature was considerably larger than the protein size, typically below 10 nm. Therefore, one can assume that the protein molecules effectively adsorb onto an atomically smooth surface (i.e., well below one nanometer) and that they are effectively immersed in oscillating shearing flow as demonstrated by the theory.

The zeta potential of the bare gold and silica/gold layers functionalized by the PAH adsorption was acquired by the streaming potential measurements carried out in a parallel-plate microfluidic channel[3]. Several separate runs were performed at various pressure differences that yielded the slope of the streaming potential vs. hydrostatic pressure difference dependence. Using this slope, the zeta potential of the substrate was calculated from the Smoluchowski equation. The pH of the PAH solution was 5.6, the bulk concentration 5 mg L^{-1} and the flow volumetric flow rate 0.35 cm^3 s^{-1}. To prevent the PAH macroion depletion, all glassware was preconditioned three times with the macroion solutions of the same concentration as that used in the experiments.

It was determined that the zeta potential for the gold/silica substrate was equal to -50 and - 40 mV, for the NaCl concentration of 1 and 10 mM, respectively and pH of 5.6.

Analogously, the zeta potential for the gold/PAH substrate was equal to 60 and 40 mV, for the NaCl concentration of 1 and 10 mM and the same pH.

3. Experimental Determination of the Impedance

Initially a series of QCM kinetic runs yielding the frequency and dissipation shifts for various overtones n (1 to 11) were performed for the particles under different ionic strengths, pHs and suspension volumetric flow rates and bulk suspension concentrations.

The primary kinetic runs acquired for the A70 and L200 particles are shown in Figures S2 and S3, respectively.

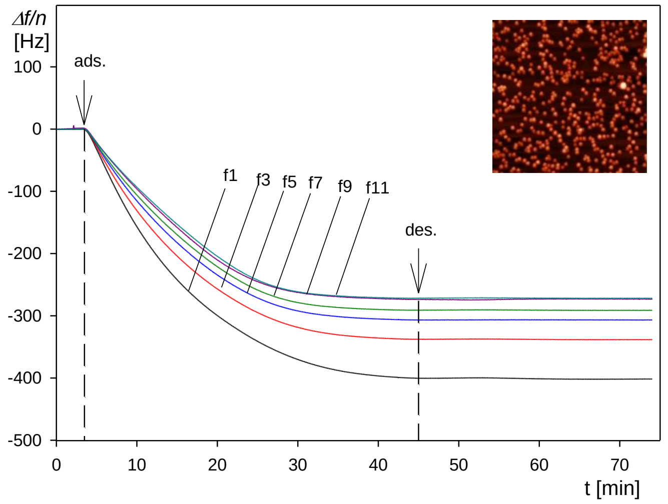

Figure S2. The primary kinetic run for the A70 particles, showing the dependence of $-\Delta f/n$ on the deposition time for the overtones 1-11: Experimental conditions: gold/silica sensor, 10 mM NaCl, pH 4, volumetric flow rate 2.5×10^{-3} cm^3 s^{-1}, bulk particle concentration 20 mg L^{-1}. The inset shows the particle layer imaged by AFM.

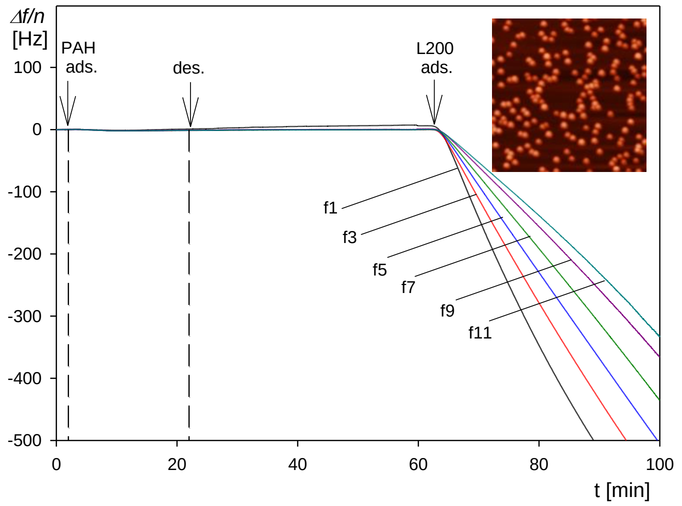

Figure S3. The primary kinetic run for the L200 particles, showing the dependence of $-\Delta f/n$ vs. deposition time for the overtones 1-11: Experimental conditions: gold/PAH sensor, 10 mM NaCl, pH 4, volumetric flow rate 2.5×10^{-3} cm^3 s^{-1}, bulk particle concentration 200 mg L^{-1}. The inset shows the particle layer imaged by AFM.

After completing each run, the real particle coverage was determined by AFM according to the above-described procedure. This parameter was used to control the precision of the kinetic runs derived for the hybrid RSA modeling[4-7], which furnished the particle mass coverage vs. the adsorption time dependencies in a quasi-continuous manner. Such a procedure, previously applied in Ref. [1], enabled a reliable determination of the impedance and other derivative functions used for the interpretation of the QCM measurements.

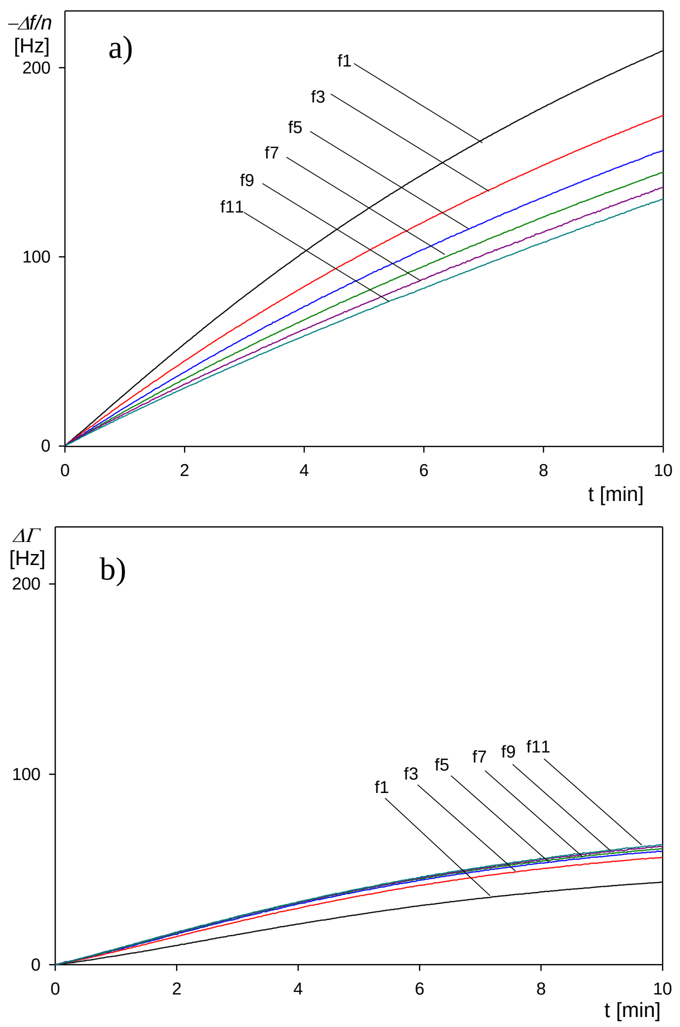

Figure S4. The short time deposition kinetic of the A73 particles derived from QCM:
a) Frequency shift $-\Delta f/n$ vs. deposition time for the overtones 1-11,
b) bandwidth shift $\Delta\Gamma$ vs. deposition time for the overtones 1-11.
Experimental conditions: gold/silica sensor, 10 mM NaCl, pH 4, volumetric flow rate 2.5×10^{-3} cm^3 s^{-1}, bulk particle concentration 20 mg L^{-1}.

The short time kinetic data derived for the A70 particles are shown in Figure S4 the dependence of the normalized frequency shifts $-\Delta f/n$ (subplot a) and the bandwidth shifts

$\Delta\Gamma = \frac{1}{2} f_0 \Delta D$ (subplot b) on the time (where f_0 is the fundamental frequency of the sensor equal to 5×10^6 Hz and ΔD is the dissipation shift). As can be seen, these dependencies only remain linear for approximately 4 minutes. Therefore, to increase the precision of the slope determination in the limit of *t* tending to zero, which is needed for the impedance determination, was calculated using a polynomial fitting procedure.

Using the QCM signals shown in Figure S4, the complex impedance components ΔZ^* can be calculated from the constitutive dependencies:

$$\mathrm{Im}(\Delta Z^*) = -\frac{\pi Z_q}{f_0}\Delta f \, , \tag{S1}$$

$$\mathrm{Re}(\Delta Z^*) = -\frac{\pi Z_q}{f_0}\Delta \Gamma \, , \tag{S2}$$

where Z_q is the acoustic impedance of quartz equal to 8.8×10^6 kg m^{-2} s^{-1}.

For a purely inertial load the impedance is given by[8-10]

$$\begin{aligned} \mathrm{Im}(\Delta Z^*) &= \omega m \tilde{n} = \omega M \, , \\ \mathrm{Re}(\Delta Z^*) &= 0 \, , \end{aligned} \tag{S3}$$

where $\omega = 2\pi f_0 n$ is the angular velocity of the sensor oscillations and M is the mass of the particle layer per unit area, referred to as the mass coverage. As mentioned above, this parameter was determined from the RSA modeling calibrated using the real particle coverage derived from AFM.

Using this inertia load impedance, i.e., ωM as a scaling variable one obtains the following expressions connecting the normalized impedance $\overline{Z}^*$ components with the frequency and the bandwidth shifts:

$$\begin{aligned} \mathrm{Im}\left(\overline{Z}^*\right) &= \frac{\Delta f(t)}{\Delta f_S(t)} = -C_s \frac{\Delta f(t)}{n\,\Gamma(t)} = \frac{M_Q(t)}{M(t)} \, , \\ \mathrm{Re}\left(\overline{Z}^*\right) &= -C_s \frac{\Delta \Gamma(t)}{M(t)} = -A(t)\,\mathrm{Im}\left(\overline{Z}^*\right) \, , \end{aligned} \tag{S4}$$

where $\Delta f_S(t) = -\frac{f_0 \omega m \tilde{n}}{\pi Z_q}$ is the frequency shift expected for a purely inertial load (referred to as the Sauerbrey shift) and

$$M_Q(t) = -\frac{C_s\,\Delta f(t)}{n} \tag{S5}$$

is the apparent QCM coverage often referred to as the ‘wet’ mass, $C_s = \frac{Z_q}{2f_0^2}$ is the Sauerbrey constant equal to 0.177 (mg m^{-2}) Hz^{-1} for $f_0 = 5 \times 10^6$ Hz and

$$A = -\frac{\Delta\Gamma(t)}{\left[\frac{\Delta f(t)}{n}\right]}\,. \tag{S6}$$

Knowing $M_Q(t)$ (derived from eq S5) and $M(t)$ (derived from RSA/AFM) one can directly calculate the dependence of the impedance components on the time using eq S4. The impedance components can also be expressed in terms of the real particle coverage by eliminating the time variable from eq S4, which can be calculated by a numerical inversion of the $M(t)$ dependence, i.e.,

$$t = M(t)^{-1}\,. \tag{S7}$$

The impedance $\overline{Z}_0^*$ components in the limit of vanishing particle coverage can be precisely determined from the following formulae:

$$\begin{aligned} \mathrm{Im}\left(\overline{Z}_0^*\right) &= \frac{\left(\frac{dM_Q}{dt}\right)}{\left(\frac{dM}{dt}\right)} = \frac{s_{lQ}}{s_l}, \\ \mathrm{Re}\left(\overline{Z}_0^*\right) &= -A_{r0}\,\mathrm{Im}\left(\overline{Z}_0^*\right), \end{aligned} \tag{S8}$$

where s_{lQ} and s_l are the corresponding slopes of the $M_Q(t)$ and $M(t)$ dependencies (fitted by second order polynomials) in the limit of a vanishing time.

To facilitate the comparison of the experimental data with theoretical modeling, the impedance components can be alternatively expressed in the following form:

$$\mathrm{Im}\left(\overline{Z}_0^*\right) = -\frac{3}{8}\left(\frac{\delta}{a}\right)^2\left(\frac{\rho}{\rho_s}\right)\frac{\Delta f}{f_0\alpha},$$

$$\mathrm{Re}\left(\overline{Z}_0^*\right) = \frac{3}{8}\left(\frac{\delta}{a}\right)^2\left(\frac{\rho}{\rho_s}\right)\frac{\Delta \Gamma}{f_0\alpha}, \tag{S9}$$

where $\alpha = \frac{\eta a \tilde{n}}{Z_q}$ is the (viscous-to-solid) impedance ratio, $\delta = \left(\frac{2v}{\omega}\right)^{1/2} = \left(\frac{v}{\pi n f_0}\right)^{1/2}$ is the viscous penetration depth and, v is the kinematic viscosity of the fluid and ρ its density.

4. Theoretical Predicitons for the Impedance and the Acoustic Ratio

Table S3. Theoretical prediction of the scaled frequency and bandwidth shifts, impedance components (calculated from eqs S9) and the acoustic ratio for neutrally buoyant particles, where $\rho/\rho_s = 1$, with a stiff attachment to the sensor. Some of these results are shown in Figures 4a, b in the main text.

a/δ	$-\Delta f/(f_0\alpha)$	$\mathrm{Im}(\overline{Z}_0^*)$	$\Delta\Gamma/(f_0\alpha)$	$\mathrm{Re}(\overline{Z}_0^*)$	$\Delta\Gamma/(-\Delta f)$
0.01	0.0029	10.9	0.000044	0.165	0.0151
0.02	0.0113	10.6	0.00034	0.319	0.0300
0.03	0.0251	10.5	0.00111	0.463	0.0442
0.04	0.0439	10.3	0.00255	0.598	0.0581
0.05	0.0675	10.1	0.00484	0.726	0.0717
0.06	0.0956	9.96	0.00810	0.844	0.0847
0.07	0.128	9.80	0.0125	0.957	0.0977
0.08	0.165	9.67	0.0180	1.05	0.109
0.09	0.205	9.49	0.0249	1.15	0.121
0.1	0.249	9.34	0.0331	1.24	0.133
0.2	0.844	7.91	0.190	1.78	0.225
0.3	1.64	6.83	0.464	1.93	0.283
0.4	2.58	6.05	0.820	1.92	0.318
0.5	3.64	5.46	1.24	1.86	0.341
0.6	4.80	5.00	1.72	1.79	0.358
0.7	6.05	4.63	2.23	1.71	0.369
0.8	7.39	4.33	2.76	1.62	0.373
0.9	8.82	4.08	3.31	1.53	0.375
1.	10.33	3.874	3.87	1.489	0.3843
1.2	13.62	3.547	5.03	1.310	0.3693
1.4	17.27	3.304	6.22	1.190	0.3601
1.6	21.28	3.117	7.43	1.009	0.3492
1.8	25.64	2.968	8.67	1.003	0.3381
2.	30.34	2.844	9.93	0.9309	0.3273
2.4	40.81	2.657	12.50	0.8138	0.3063
2.8	52.67	2.519	15.14	0.7242	0.2874
3.2	65.91	2.414	17.83	0.6566	0.2720
3.6	80.52	2.329	20.57	0.5952	0.2555
4.	96.50	2.262	23.35	0.5473	0.2420

5. Estimate of the QCM Contact Stiffness

We assume that "rocking" (i.e., turning about the point of contact with the resonator) of the adhesive particle of radius a is opposed by the (elastic) contact torque originating in the small contact region[11]:

$$L_c^{(e)} = \kappa_b a^2 \frac{\Delta u}{a}, \quad \text{(S10)}$$

where $\Delta u = u_p - u_0$ is the displacement of the particle's center with respect to that of the resonator, such that $\Delta u/a$ is the turning angle. The bending stiffness κ_b (in units of force/length), can be approximated by[11]:

$$\kappa_b \approx 6\pi W = \frac{4E_c r_c^3}{3a^2} \approx 2\kappa_s \left(\frac{r_c}{a}\right)^2, \quad \text{(S11)}$$

where W is the work of adhesion per unit area, E_c is an *effective modulus* of the contact region (of the order of the Young's modulus, depending on properties of both the particle and the substrate), and r_c is the radius of the contact area; the shear stiffness satisfies $\kappa_s \gg \kappa_b$, implying upon oscillations of the sensor, the particle would rather exhibit "rocking" than "sliding".

The hydrodynamic torque (with respect to the point of contact) exerted on the small enough adsorbate can be estimated to the leading approximation as (see the Section "Small-particle limit" in the main text) as:

$$\frac{L_c^{(h)}}{\eta a^2 v_0} \approx \mathcal{C}\left(\frac{a}{\delta}\right), \quad \text{(S12)}$$

Where the dimensionless constant $\mathcal{C} = 13.98\pi$ and $\delta = \sqrt{2\nu/\omega}$ is the viscous penetration depth.

Equating $L_c^{(e)} \approx L_c^{(h)}$ and using the eqs S10-S12, we can estimate the velocity differential scaled with the velocity of the resonator, $\Delta v/v_0$, as:

$$\frac{\Delta v}{v_0} = \frac{\omega \Delta u}{v_0} = \frac{\mathcal{C}\omega a^2 \eta}{6\pi W \delta} \approx 1.6\, \frac{\omega^{3/2} a^2 (\eta\rho)^{1/2}}{W}. \quad \text{(S13)}$$

Notice that the ratio $\Delta v/v_0$ is equivalent to $\Delta u/A$, i.e., a displacement normalized by the amplitude of the resonator oscillations, $A = v_0/\omega$, which is typically <1 nm. We assume that a stiff contact complies with $\Delta v/v_0 < 1$. Clearly, $\Delta v/v_0$ scales as a^2 and $\omega^{3/2}$, indicating that the stiff contact assumption in QCM experiments may not hold for large enough adsorbates and/or high enough oscillation frequency. These dependencies are in qualitative agreement with our experimental findings, showing deviation from the theory at higher frequencies and for larger adsorbates.

The work of adhesion W cannot be easily estimated from the first principles due to occurrence of the electrical double layers and nonhomogeneous sensor roughness. It is possible, however, to estimate its value based on the experimental observations using the approximate criterion $\Delta v/v_0 \approx 1$ in eq S13 as the threshold of sustaining a stiff contact. The experimental data (see Figures 4 a,b in the main text) demonstrate that positively charged amidine polystyrene particles with radius $a = 36$ nm (A70) adsorbing onto a negatively charged SiO_2 resonator from an aqueous solution, fail to maintain stiff attachment starting from the 9th overtone, resulting in $W(\mathrm{PS/SiO_2}) \approx 11\ \mathrm{mJ/m^2}$. The frequency response of smaller amidine polystyrene particles with $a = 13$ nm (A26) adsorbing onto SiO_2 sensor suggests that the stiff contact assumption holds for all tested overtones 1-11.

For larger negatively charged sulfonate polystyrene particles with radius $a \approx 105$ nm (A200) adsorbing onto a positively charged gold sensor, the experimental findings show that stiff contact is sustained only up to the 3rd overtone. Using the condition $\Delta v/v_0 = 1$ in eq S13 for $n = 5$ yields the estimate $W(\mathrm{PS/Gold}) \approx 34\ \mathrm{mJ/m^2}$.

Given the estimated work of adhesion in both cases, we can find the contact area radius from eq S11 as $r_c = (9\pi W a^2/2E_c)^{1/3} \approx 1.7$ nm and $r_c \approx 9.9$ nm, for A70 and A200 particles, respectively, where in both cases we used the effective modulus of the contact $E_c \approx$ 5 GPa[8].

References

1. Adamczyk, Z.; Sadowska, M.; Żeliszewska, P. Applicability of QCM-D for quantitative measurements of nano- and microparticle deposition kinetics: Theoretical modeling and experiments. *Anal. Chem*. **2020**, 92, 15087–15095.

2. Adamczyk, Z.; Sadowska, M.; Nattich-Rak, M.; Quantifying nanoparticle layer topography: Theoretical modeling and atomic force microscopy investigations. *Langmuir* **2023**, 39, 15067-15077.

3. Morga, M.; Adamczyk, Z. Monolayer of cationic polyelectrolytes on mica- Electrokinetic studies. *J. Colloid Interface Sci.* **2013**, 407, 196-204.
4. Adamczyk, Z. Particles at interfaces: Interactions, deposition, structure. Elsevier. **2017**.
5. Adamczyk, Z. Kinetics of diffusion-controlled adsorption of colloid particles and proteins. *J. Colloid Interface Sci*. **2000**, 229, 477-489.
6. Schaaf, P.; Talbot, J. Surface exclusion effects in adsorption processes. *J. Chem. Phys.* **1989**, 91, 4401–4409.
7. Talbot, J.; Tarjus, G.; Van Tassel, P.R.; Viot, P. From car parking to protein adsorption: an overview of sequential adsorption processes. *Colloids Surf. A Physicochem. Eng. Asp.* **2000**, 165, 287–324.
8. Tarnapolsky, A.; Freger, V. Modeling QCM-D response to deposition and attachment of microparticles and living cells. *Anal. Chem*. **2018**, 90, 13960–13968.
9. Meléndez, M.; Vázquez-Quesada, A.; Delgado-Buscalioni, R. Load impedance of immersed layers on the quartz crystal microbalance: A comparison with colloidal suspensions of spheres. *Langmuir* **2020**, 36, 9225−9234.
10. Delgado-Buscalioni, R. Coverage effects in quartz crystal microbalance measurements with suspended and adsorbed particles. *Langmuir* **2024**, 40, 580-593.
11. Dominik, C.; Tielens, A. G. G. M. Resistance to Rolling in the Adhesive Contact of Two Elastic Spheres. *Philos. Mag. A Phys. Condens. Matter, Struct. Defects Mech. Prop.* **1995**, *72* (3), 783–803.
